# Study of the Role of the Tyrosine Kinase Receptor MerTK in the Development of Kidney Ischemia-Reperfusion Injury in RCS Rats

**DOI:** 10.3390/ijms222212103

**Published:** 2021-11-09

**Authors:** Thomas Pelé, Sebastien Giraud, Sandrine Joffrion, Virginie Ameteau, Adriana Delwail, Jean-Michel Goujon, Laurent Macchi, Thierry Hauet, Fatima Dkhissi, Omar Benzakour

**Affiliations:** 1 INSERM U1082 (IRTOMIT), 86000 Poitiers, France; thomas.pele9@gmail.com (T.P.); giraudseb@yahoo.fr (S.G.); joffrion.sandrine@gmail.com (S.J.); virginie.ameteau@univ-poitiers.fr (V.A.); j.m.goujon@chu-poitiers.fr (J.-M.G.); laurent.macchi@chu-poitiers.fr (L.M.); thierry.hauet@univ-poitiers.fr (T.H.); fatima.dkhissi@univ-poitiers.fr (F.D.); 2 Service de Biochimie, CHU Poitiers, 86000 Poitiers, France; 3 Faculté de Médecine et de Pharmacie, Université de Poitiers, 86000 Poitiers, France; 4 Plateforme d’Imagerie de l’Université de Poitiers et CNRS ERL 7003/EA 7349, Université de Poitiers, 86000 Poitiers, France; adriana.delwail@univ-poitiers.fr; 5 Service d’Anapathomopathologie, CHU Poitiers, 86000 Poitiers, France; 6 Service d’Hématologie, CHU Poitiers, 86000 Poitiers, France

**Keywords:** ischemia-reperfusion, MerTK, microparticles, inflammation, phagocytosis, RCS rats

## Abstract

Renal ischaemia reperfusion (I/R) triggers a cascade of events including oxidative stress, apoptotic body and microparticle (MP) formation as well as an acute inflammatory process that may contribute to organ failure. Macrophages are recruited to phagocytose cell debris and MPs. The tyrosine kinase receptor MerTK is a major player in the phagocytosis process. Experimental models of renal I/R events are of major importance for identifying I/R key players and for elaborating novel therapeutical approaches. A major aim of our study was to investigate possible involvement of MerTK in renal I/R. We performed our study on both natural mutant rats for MerTK (referred to as RCS) and on wild type rats referred to as WT. I/R was established by of bilateral clamping of the renal pedicles for 30′ followed by three days of reperfusion. Plasma samples were analysed for creatinine, aspartate aminotransferase (ASAT), lactate dehydrogenase (LDH), kidney injury molecule -1 (KIM-1), and neutrophil gelatinase-associated lipocalin (NGAL) levels and for MPs. Kidney tissue damage and CD68-positive cell requirement were analysed by histochemistry. monocyte chemoattractant protein-1 (MCP-1), myeloperoxidase (MPO), inducible nitric oxide synthase (iNOS), and histone 3A (H3A) levels in kidney tissue lysates were analysed by western blotting. The phagocytic activity of blood-isolated monocytes collected from RCS or WT towards annexin-V positive bodies derived from cultured renal cell was assessed by fluorescence-activated single cell sorting (FACS) and confocal microscopy analyses. The renal I/R model for RCS rat described for the first time here paves the way for further investigations of MerTK-dependent events in renal tissue injury and repair mechanisms.

## 1. Introduction

Renal ischemia-reperfusion (I/R) is a sequence of events including oxidative stress, cell death, and inflammation that may cause organ failure and is unavoidable during renal transplantation [1,2,3,4,5]. Dying cells may release damage-associated molecular patterns (DAMPs) that are mainly pro-inflammatory factors responsible for leukocyte recruitment as well as microparticle (MPs) [6,7]. At the site of tissue injury, neutrophils may release their DNA contents that act as neutrophil extracellular traps (NET) and induce surrounding cell necrosis, enhancing the inflammatory process [8,9,10]. Macrophages, through reactions between Nitric Oxyde (NO) and Reactive Oxygen Species (ROS), release peroxnitrites, which are cytotoxic products that contribute to cell damage while recruited leukocytes by phagocytosing cellular debris contribute to the repair process [11,12,13,14].

Microparticles (MPs) are 0.1 µm to 1 µm diameter vesicles that are characterized by phosphatidylserine (PS) exposure on the external leaflet membrane. They are characterized by both PS exposure on their external surface and by the presence of markers reflecting their cellular origin [15,16]. Since they may act as vehicles for intercellular communication, by transferring their contents to target cells, by receptor-mediated signaling or by releasing of soluble signaling molecules, MPs are believed to play an important role in several processes including hemostasis, inflammation, vascular tone, and tumor growth [17,18]. Under basal conditions, most MPs detected in peripheral blood are derived from platelets (PMPs) or leukocytes (LMPs) or endothelial cells (EMPs) [17]. Following hepatic ischemia reperfusion, the levels of circulating LMPs, PMPs, and EMPs increase [19]. Circulating MPs expose PS that acts as an eat-me signal and that is recognized by specific receptors expressed by phagocytes. The tyrosine kinase receptor MerTK expressed by macrophages plays an important role in MP phagocytosis [18].

MerTK is a tyrosine kinase receptor of the TAM (Tyro3, Axl, and MerTK) family. MerTK is expressed by many cells including endothelial cells and monocytes/macrophages and is also expressed within the kidney by glomerular cells [20,21,22]. MerTK is a key player in apoptotic cell clearance by phagocytosis; its ligand, the anticoagulant vitamin K-dependent factor protein S, bridges PS exposed on the plasma membrane of apoptotic cells with the MerTK receptor on the surface of phagocytes, enabling phagocytosis to take place [23,24,25]. The absence of a functional MerTK protein leads to a defect in phagocytosis that has been associated with several pathologies such as impaired spermatogenesis, photoreceptor degeneration or autoimmune disease [24,26,27,28,29]. MerTK expressed by macrophages may also act as a negative regulator of inflammation, as impaired MerTK expression leads to an increase in pro-inflammatory mediators such as interleukin-6 (IL-6), monocyte chemoattractant protein-1 (MCP-1), and tumor necrosis factor-α (TNF-α) [30]. For the kidney, a protective role of MerTK in nephrotoxic serum-induced nephritis was described but its possible roles, either in vitro or in vivo, in renal I/R have not been studied as yet [20]. RCS rat models, which are natural mutants for MerTK (RCS) vs. wild type rats (WT), were previously used mainly for studying the effects of MerTK on retina degeneration linked to a defect in photoreceptor outer segment phagocytosis [31,32,33,34,35].

In order to study the putative role of a functional MerTK receptor in the renal I/R sequence, we describe herein for the first time the use of an RCS rat model for the study of renal I/R. In this model, we monitored many parameters linked to renal function, inflammation, circulating MPs as well as in vitro assays of phagocytosis.

## 2. Results

### 2.1. Renal I/R Induces an Increase in Several Plasma Biomarkers of Injury in Both WT and RCS Rats

PCR and western blot experiments described in supplementary data online (Figure S1) confirmed that RCS rats that were used in the present study, noted RCS hereafter, do not express a full functional MerTK protein.

For each of the groups and subgroups defined in the methods section, the plasma levels of the following known biomarkers of renal I/R injury: Creatinine, Urea, aspartate aminotransferase (ASAT), lactate dehydrogenase (LDH), kidney injury molecule -1 (KIM-1), and neutrophil gelatinase-associated lipocalin (NGAL), were determined seven days before surgery (D-7, baseline level), on day 1 (D1), and on day 3 (D3) post-I/R.

**Figure 1:**
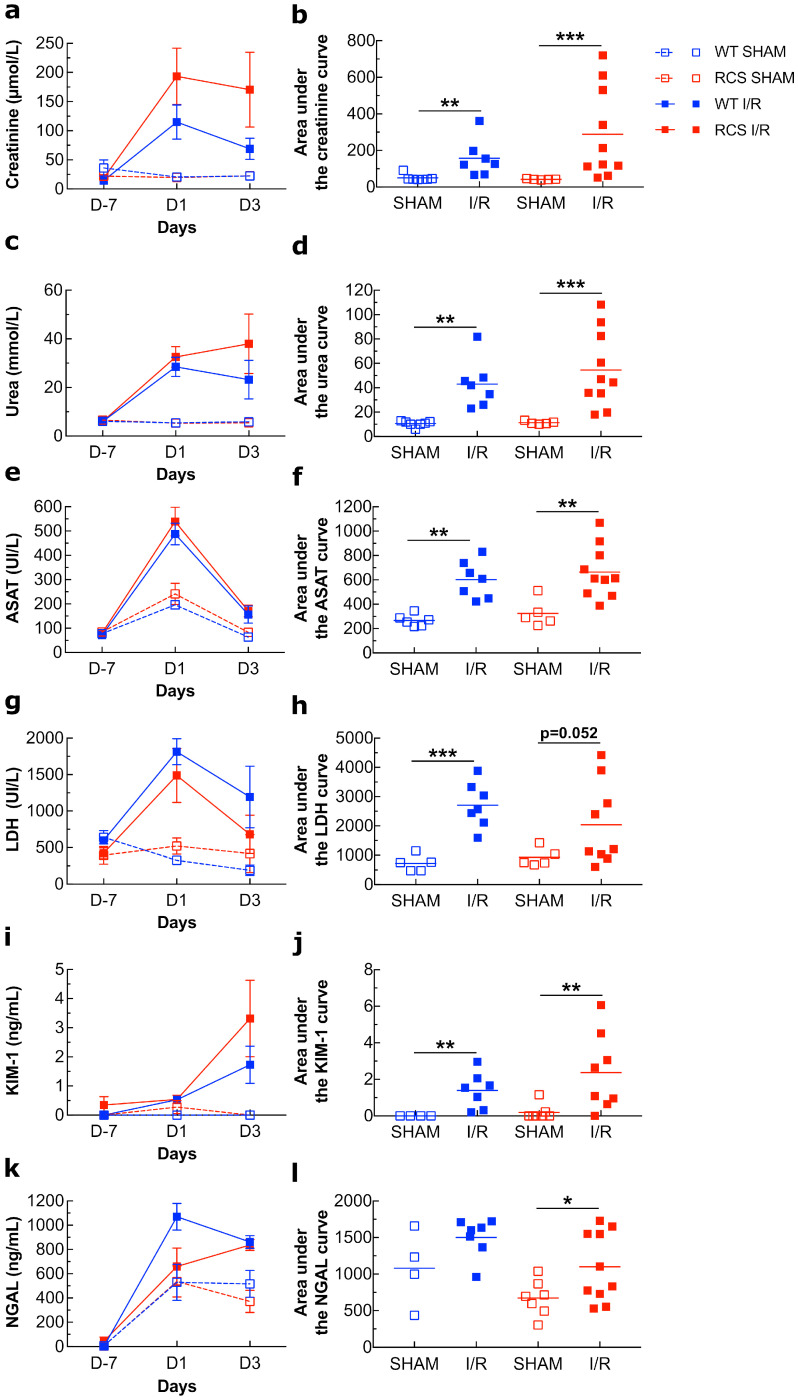
Plasma levels of creatinine, urea, ASAT, LDH, KIM-1, and NGAL in both WT and RCS under SHAM or I/R conditions on day 1 and day 3 post-reperfusion. Plasma from both WT or RCS rats that were not submitted to surgery (D-7), were submitted to surgery without renal pedicle clamping (SHAM) or were submitted to a 30-min renal pedicle clamping (I/R) was analyzed on day 1 (D1) and on day 3 (D3) post-reperfusion or post-surgery for creatinine (**a**), urea (**c**), ASAT (**e**), LDH (**g**), KIM-1 (**i**) or NGAL (**k**) levels. Creatinine, urea, LDH, and ASAT levels were measured using a Cobas C701 automatic analyzer. Plasma NGAL and KIM-1 levels were determined by sandwich ELISA. For each marker and each condition, five to 10 rats were used; data are presented as means ± SEM. The area under the curve (from D-7 to D3) for each marker and each condition was calculated using R-studio software and is represented in panels (**b**) (creatinine), (**d**) (urea), (**f**) (ASAT), (**h**) (LDH), (**j**) (KIM-1), and (**l**) (NGAL); lines represent the mean for each condition. The Mann–Whitney test was used for determining the *p* values. *: *p* < 0.05; **: *p* < 0.01; ***: *p* < 0.005.

Figure 1a,b show that compared to the control (RCS: 20.2 µmol/L ± 1.0; WT: 21 µmol/L) or to the SHAM subgroup (RCS: 19.8 µmol/L ± 0.6; WT: 20.75 µmol/L ± 0.4), the I/R sequence induced on day 1 (RCS: 193.4 µmol/L ± 48.3; WT: 115 µmol/L ± 29.4) and to a lesser extent on day 3 post-I/R sequence, a sharp increase in the creatinine plasma concentrations. A similar pattern was observed for Urea (Figure 1c,d), ASAT (Figure 1e,f), LDH (Figure 1g,h), and NGAL (Figure 1k,l) plasma concentrations. For NGAL plasma concentrations (Figure 1k,l) the increase induced by the I/R sequence, on both day 1 and to a lesser extent on day 3, was weaker than that observed for the above markers. For KIM-1 plasma concentrations (Figure 1i,j) the increase induced by the I/R sequence was observed on day 3 post-reperfusion. In comparison with the SHAM group, for some markers, the fold increase of the I/R groups was very different between RCS and WT. For instance, the creatinine plasma concentration increased by about 10-fold in RCS and by 5.6-fold in the WT group; LDH increased by 6-fold in RCS and by 9-fold in WT.

**Figure 2:**
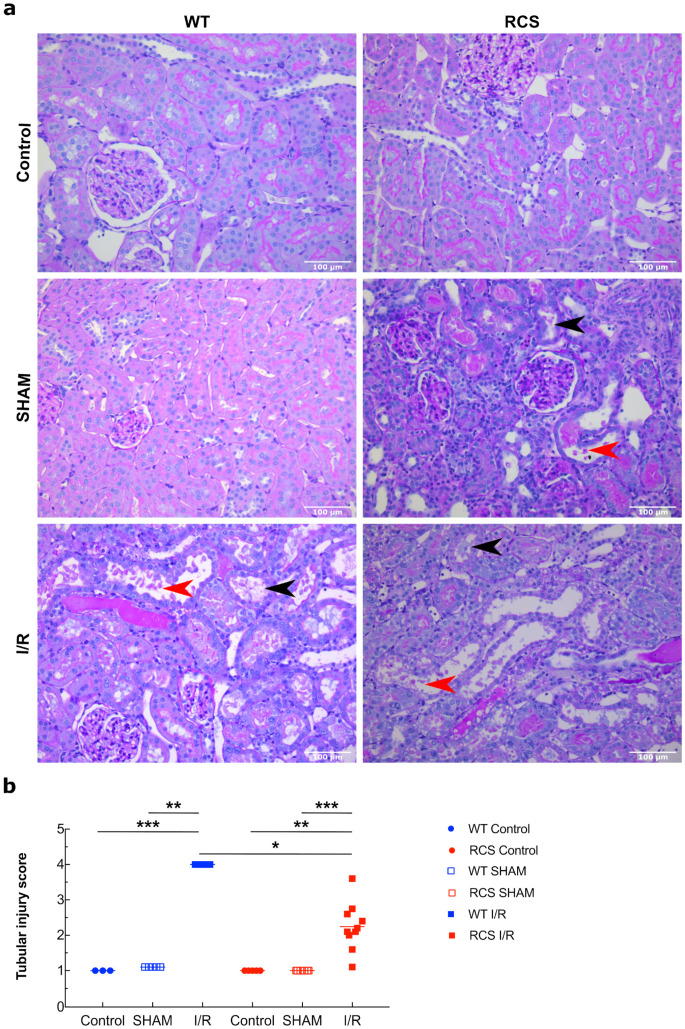
Histological analysis of kidney sections from both WT and RCS in the Control, SHAM, and I/R groups on day 3 post-reperfusion. Kidney sections from the Control, SHAM or I/R groups (three days after surgery or reperfusion) were stained with PAS coloration; the presence and the extent of tubular injury were examined and scored under the microscope (x200). Panel (**a**) represents the typical structure observed under each condition; the red arrows indicate cellular debris in the lumen while the black arrows indicate brush border loss; the scale bar represents 100 µm. In panel (**b**), the score of tubular injury from five to 13 rats from each condition was averaged and is presented; lines represent the mean for each condition. The Mann–Whitney test was used for determining the *p* values. *: *p* < 0.05; **: *p* < 0.01; ***: *p* < 0.005.

Histological analyses of kidney sections in the experimental groups (WT and RCS) and subgroups are depicted in Figure 2. They show that at three days post-reperfusion for the I/R group, tissue damage was mainly localized within the tubules region as illustrated by the loss of the brush border (black arrow) and the presence of cellular debris in the lumen of the tubules (red arrow) (Figure 2a). Such tissue damage was significantly less (*p* < 0.05) pronounced in RCS compared to WT (Figure 2b).

Altogether, data from Figure 1 and Figure 2 suggest that the I/R sequence induced alterations to both renal function as determined from the observed changes in the plasma levels of several markers and renal tissue as determined from histological analyses. These results show that the absence of a functional MerTK receptor has an impact on the extent of the I/R-induced changes in the plasma levels of some markers such as creatinine and LDH and in renal histological damage. Such alterations and injuries may be triggered by inflammatory molecules and may in turn trigger inflammatory events.

### 2.2. Renal I/R Sequence Triggers Multiple Inflammatory Responses in Both RCS and WT Rats

**Figure 3:**
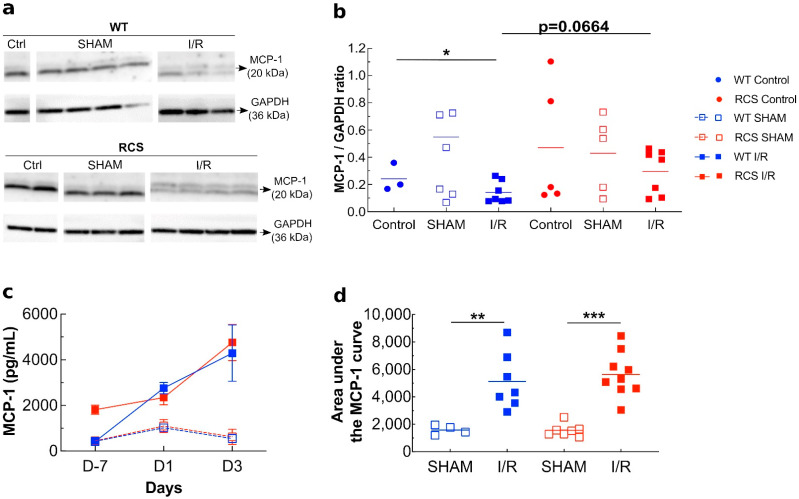
Kidney and plasma levels of MCP-1 in both WT and RCS rats under control, SHAM, and I/R conditions on day 1 or day 3 post-reperfusion. In panel (**a**), kidney tissue lysates from both WT or RCS rats that were not submitted to surgery (Control), were submitted to surgery without renal pedicle clamping (SHAM) or were submitted to a 30-min renal pedicle clamping followed by three days reperfusion (I/R) were analyzed by western blotting for the presence of the MCP-1 protein (20 kDa); protein loads are shown at the bottom of each western blot. In panel (**b**), the intensity of MCP-1 bands was normalized to the GAPDH protein and are presented; three to eight rats were used for each condition. In panel (**c**), plasma MCP-1 levels, at 1- and 3-days post-surgery or post-reperfusion, determined by ELISA are presented. The area under the curve relative to panel (**c**) was calculated using R-studio software and is represented in panels (**d**); four to nine rats were used; lines represent the mean for each condition. The Mann–Whitney test was used for determining the *p* values. *: *p* < 0.05; **: *p* < 0.01; ***: *p* < 0.005.

We investigated possible effects of a renal I/R sequence, in our experimental group (RCS and WT) and subgroups (control, SHAM and renal I/R), on the inflammatory process by monitoring possible changes in the expression level of a major chemoattractant/pro-inflammatory molecule: MCP-1. Figure 3a represents western blot analysis of tissue kidney lysate using anti-MCP-1 antibody. Its quantification (Figure 3b) shows that the renal I/R sequence induced a significant decrease in renal tissue MCP-1 expression in WT rats only. As compared to control or SHAM subgroups, the I/R sequence induced a significant increase in MCP-1 plasma levels, the extent of which differed between WT and RCS rats (Figure 3c). For example, as compared to the SHAM group, in the I/R group at D3, MCP-1 plasma levels increased by 6.8-fold in WT and by 8.7-fold in RCS rats (Figure 3c,d).

**Figure 4:**
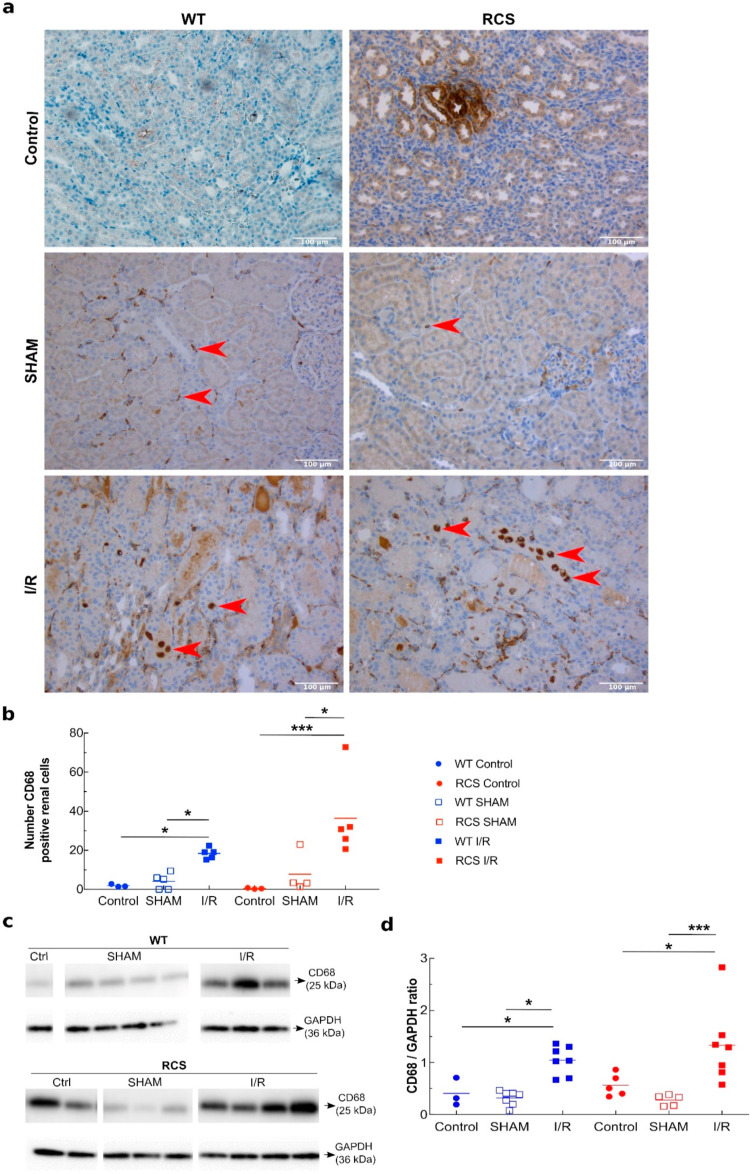
CD68-positive leukocyte recruitment within the kidney following the I/R sequence in both WT and RCS rats on day 3 post-reperfusion. Panel (**a**) represents typical structures observed by immunohistology analysis of kidney sections from the Control, SHAM or I/R groups in both WT and RCS rats on day 3 post- surgery or post-reperfusion using a monoclonal anti-CD68 antibody. The red arrows indicate CD68-positive leukocytes; the scale bar represents 100 µm. Panel (**b**) represents the numbers of CD68-positive cells scored under the microscope (×200); three to five rats were used for each condition. In panel (**c**), kidney tissue lysates from both WT or RCS rats that were not submitted to surgery (Control), were submitted to surgery without renal pedicle clamping (SHAM) or were submitted to a 30-min renal pedicle clamping followed by three days of reperfusion (I/R) were analysed by western blotting for the presence of the CD68 protein (25 kDa); GAPDH protein bands are shown at the bottom of each western blot (representative western blot). In panel (**d**), the intensity of CD68 bands normalised to the GAPDH protein are presented using 4–7 rats for each condition; lines represent the mean for each condition. The Mann–Whitney test was used for determining the *p* values. *: *p* < 0.05; **: *p* < 0.01; ***: *p* < 0.005.

**Figure 5:**
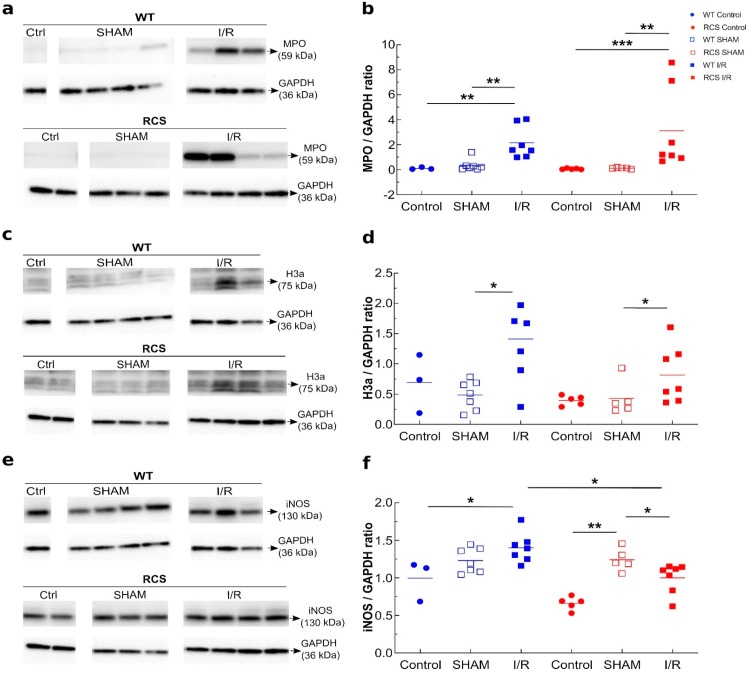
Renal tissue MPO, H3a, and iNOS protein expression in both WT and RCS rats under Control, SHAM and I/R conditions on day 3 post-reperfusion. Kidney tissue lysates from both WT or RCS rats that were not submitted to surgery (Control), were submitted to surgery without renal pedicle clamping (SHAM) or were submitted to a 30-min renal pedicle clamping were analysed by western blotting on day 3 post-surgery or post-reperfusion (I/R) for the presence of MPO (59 kDa) panel (**a**), H3a (75 kDa) panel (**c**) or iNOS (130 kDa) panel (**e**). GAPDH protein bands are shown at the bottom of each western blot (representative western blots). In panels (**b**,**d**,**f**), the intensity of MPO, H3a or iNOS bands normalized to the GAPDH protein are presented; lines represent the mean for each condition. Statistical analysis of data from five to nine rats for each group was performed using the Kruskal–Wallis test followed by Dunn’s test. The Mann–Whitney test was used for determining the *p* values. *: *p* < 0.05; **: *p* < 0.01; ***: *p* < 0.005.

MCP-1 is a chemoattractive that may trigger macrophage/neutrophil leukocyte recruitment. To deepen our study, we next investigated, in histochemistry experiments using an anti-CD68 antibody, the recruitment within the kidney of CD68-positive cell (Figure 4a). Figure 4b shows that as compared to the control, for I/R- and to a much lesser extent for SHAM- subgroups, CD68 positive cells were detected within the kidney for both RCS and WT groups. Western blot analysis of kidney tissue lysates using anti-CD68 antibody confirmed a substantial increase in the CD68 signal following the I/R sequence in both RCS and WT groups (Figure 4c,d). To ascertain and further characterize this macrophage/neutrophil phenotype recruitment following the I/R sequence within the kidney, we assessed in kidney tissue lysates the myeloperoxidase (MPO) enzyme as a marker of activated neutrophils, the inducible nitric oxide synthase (iNOS) enzyme as a marker of type-I inflammatory macrophages as well as the presence of histone H3A as a marker of NETosis (neutrophil extracellular traps). Figure 5 shows that the renal tissue expression of MPO (Figure 5a,b) and H3A (Figure 5c,d) was significantly increased by the I/R sequence in both the RCS and WT groups. However, the renal tissue expression of iNOS was only increased the WT group (Figure 5e,f).

Altogether, data from Figure 3, Figure 4 and Figure 5 suggest that in RCS rats, the I/R sequence triggered multiple inflammatory responses ranging from an increase in MCP-1 chemoattractant expression/release, macrophage/neutrophil phagocytic cells recruitment and the activation of NETosis. These results show that the absence of functional MerTK has little impact on the plasma expression of the MCP-1 chemoattractant marker (despite an increase in tissue MCP-1) or on the CD68-positive phagocytic cell recruitment in renal tissue or on the expression of activated macrophage/neutrophil markers. Therefore, the possible implication of MPs (as apoptotic targets) in this model was further investigated.

### 2.3. Effects of Renal I/R on MP Levels and Monocyte Phagocytic Activity in WT and RCS Rats

**Figure 6:**
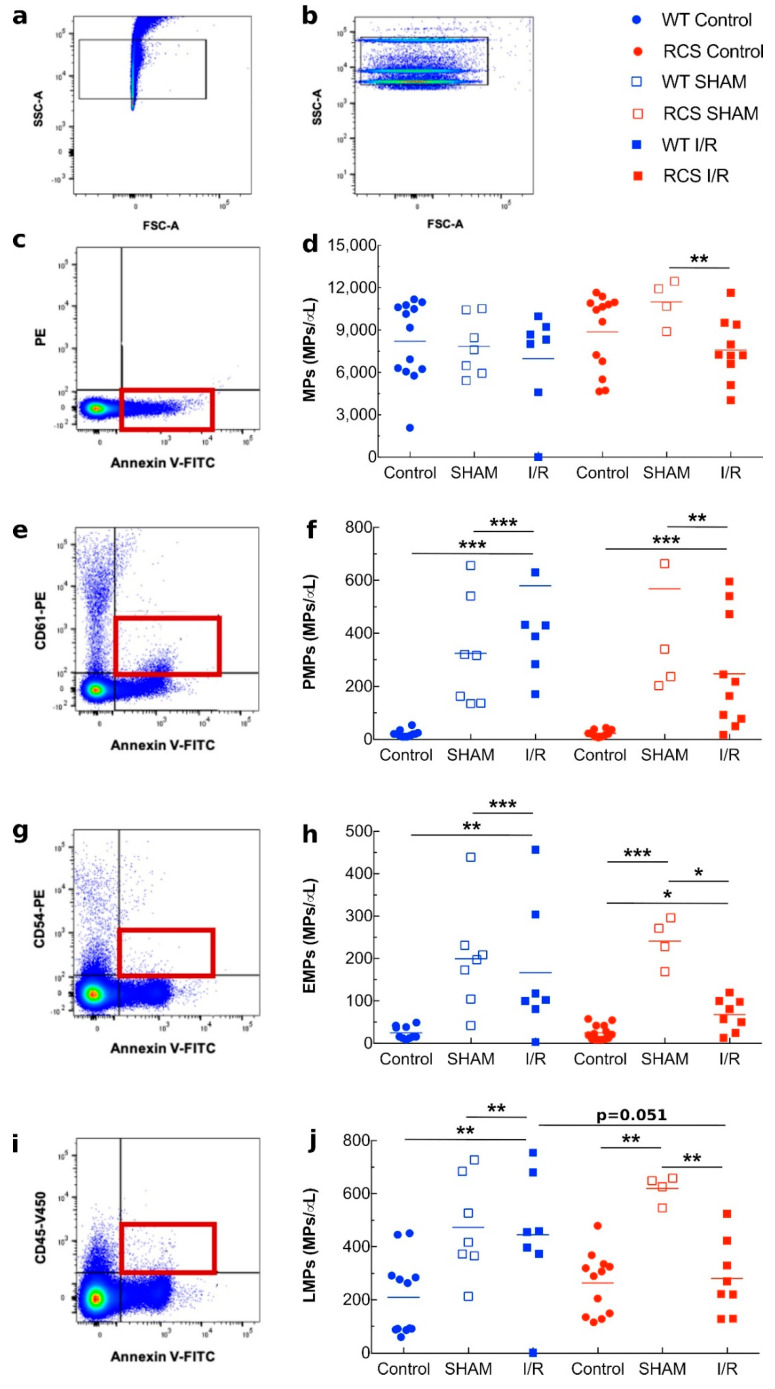
Identification of plasma MPs by flow cytometry in both WT and RCS rats under Control, SHAM, and I/R conditions on day 1 post-reperfusion. Panels (**a**,**b**) illustrate the use of Megamix-plus SSC for the settings of MP quantification by FACS analysis using a BDVerse flow cytometer. The characterization of total MPs (Annexin-V positives) from platelet-poor plasma (obtained at the control time, on day 1 after surgery without ischemia (SHAM), and on day 1 after I/R) was performed by FACS analysis and is illustrated in panel (**c**). The characterisation of cell-specific MPs was performed using annexin-V labelling in combination with anti-CD61 antibody for platelets MPs (PMPs). Panel (**e**), anti-CD54 antibody for endothelial MPs (EMPs) Panel (**g**) or anti-CD45 antibody for leukocytes MP (LMPs) Panel (**i**). For each condition, the concentration of MPs per μL of plasma was determined from four to 10 samples and is represented in panels (**d**,**f**,**h**,**j**); lines represent the mean for each condition. The Mann–Whitney test was used for determining the *p* values. *: *p* < 0.05; **: *p* < 0.01; ***: *p* < 0.005.

We next conducted flow cytometer experiments with an aim to detect and characterize MPs in Platelet Poor Plasma (PPP) following the I/R sequence. We used a variety of antibodies to determine the cellular origin of these MPs: CD61 for platelets (PMPs), CD54 for endothelial cells (EMPs), and CD45 for leukocytes (LMPs). Figure 6a illustrates how using a mix of beads of defined sizes, a size/granulometry gate was determined to locate MP populations (Figure 6a). This gate was used throughout our study (Figure 6b). Figure 6c,d, showed that as compared to the SHAM subgroup, three days post the I/R sequence, a significant decrease in the Annexin-V positive MPs level was observed in RCS but not in WT rats. As illustrated in Figure 6e,f, the PMP level showed no significant variations between RCS and WT under all the experimental conditions (control, SHAM, I/R). Figure 6g,h showed that for RCS but not for WT rats, the I/R sequence led to a substantial decrease in EMPs levels. Figure 6i,j showed that the I/R sequence led to a decrease in LMP levels in RCS but not in WT rats. For the levels of total MPs, PMPs, EMPs, and LMPs, there were significant changes between the post-surgery groups (SHAM and I/R) and the pre-surgery groups (Control). Interestingly, under the I/R condition, the LMP levels seemed more pronounced in WT as compared to RCS rats.

**Figure 7:**
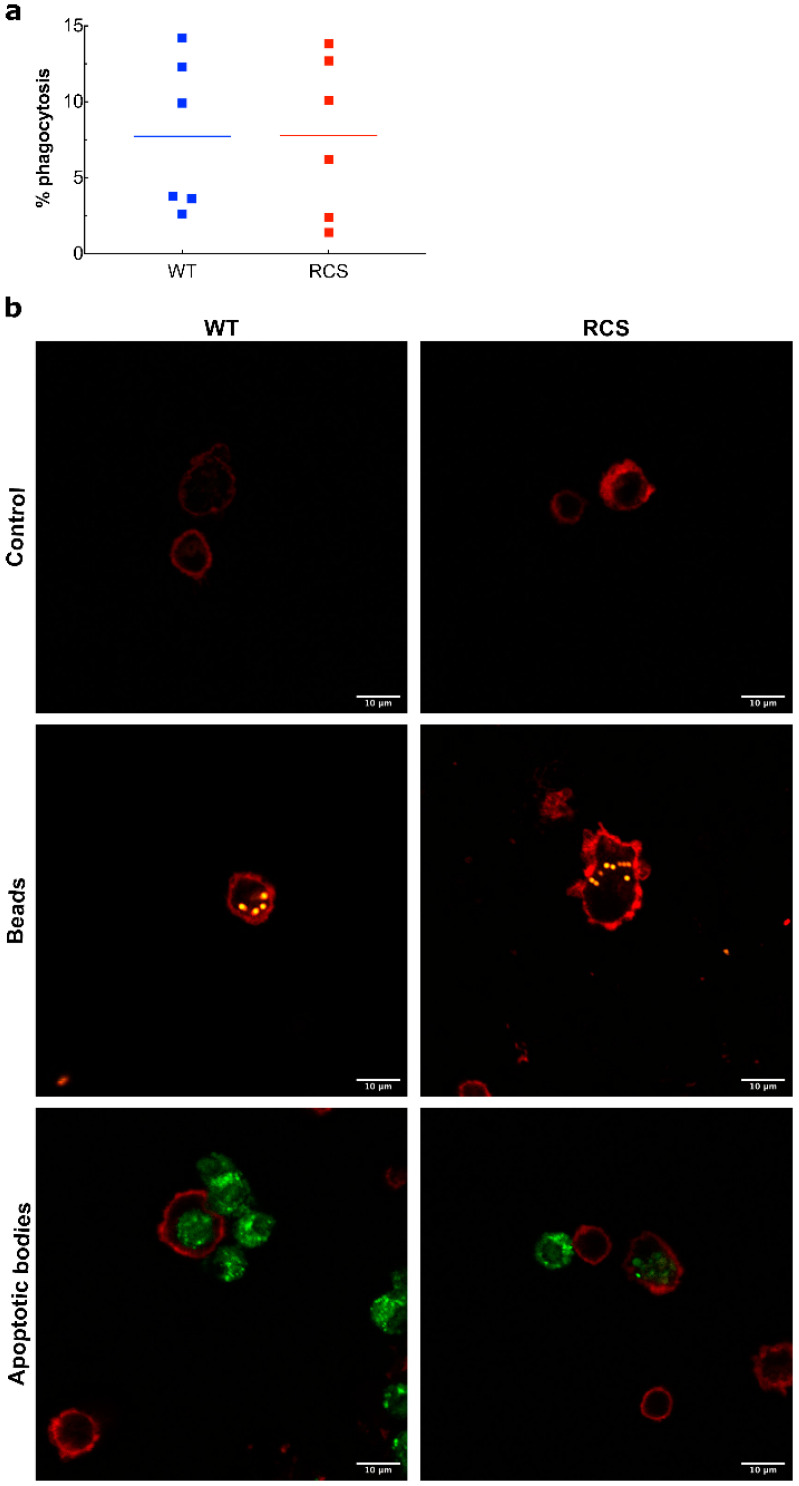
In vitro phagocytosis activity of monocytes isolated from both WT and RCS rats. Apoptotic bodies were obtained from cultured rat kidney cells (NRK-52 cells) that were exposed to 100 μM H_2_O_2_ for 3 h. NRK-52 cell apoptotic bodies were labelled with CMF-DA green tracker and used in the phagocytosis assay. Monocytes isolated from WT or RCS rat blood samples were exposed to NRK-52 cell apoptotic bodies or to fluorescent latex beads (positive control) for 9 h. Monocytes were then labelled with fluorescent (red) anti-CD45 Phycoerythrin (PE) antibody, and their ability to bind or to internalize NRK-52 cell apoptotic bodies or fluorescent latex beads was analysed by flow cytometry using an FACS BD Acuri C6 in panel (**a**) (six rats for each condition, lines represent the mean for each condition) or by confocal microscopy using an Olympus FV1000 confocal microscope in panel (**b**) (four rats for each condition). In parallel experiments, 2 µm fluorescent latex beads were used to visualise the ability of monocytes to bind or internalize latex beads and are represented in (**b**). Scale bars represent 10 µm.

The data above demonstrated that the renal I/R sequence led to an inflammatory response and to macrophage/neutrophil recruitment in both WT and RCS. Therefore, we studied the phagocytic activity of monocytes, isolated from blood collected from RCS or WT rats, towards apoptotic bodies (characterized by Annexin-V expression). Apoptotic bodies were derived from cultured NRK-52 cells that were exposed to H_2_O_2_ to mimic oxidative stress conditions. The percentage of monocytes that internalized apoptotic bodies was analyzed by flow cytometry and is expressed as monocyte phagocytosis activity (Figure 7a) and was also visualized by confocal microscopy (Figure 7b). Monocytes derived from RCS or WT rats exhibited phagocytic activity towards NRK-52 cell apoptotic bodies with a similar efficiency (7.77% for RCS and 7.73% for WT). In confocal microscopy analysis, monocytes were labelled with fluorescent (red) anti-CD45 antibody, and their ability to bind or internalize fluorescent (orange) labeled NRK-52 apoptotic bodies was visualized with CMF-DA tracker (green). Confocal microscopy analysis depicted in Figure 7b confirms that monocytes derived from RCS or WT rats were able to bind to and phagocytize NRK-52 apoptotic bodies. In our model under our experimental settings, the absence of a functional MerTK receptor did not seem to significantly impact the leucocyte phagocytosis capacity.

## 3. Discussion

The renal I/R sequence, which occurs during renal transplantation, leads to oxidative stress, cell death, and to an acute inflammatory cascade characterized by the release of many inflammatory molecules and the recruitment of inflammatory/phagocyte cells at the site of tissue injury [12]. Repair mechanisms including anti-inflammatory molecule release, leucocyte recruitment, and phagocytosis of MPs or cell debris may contribute to hemostatic recovery. Several animal models and numerous experimental set-ups were developed for studying renal I/R. However, no single animal model or experimental set-up enables us to study at once all the physio-pathological aspects of renal I/R. Therefore, additional studies with animal models evaluating novel parameters may contribute towards a better understanding of the molecular and cellular mechanisms underlying tissue injury and repair during renal I/R.

The tyrosine kinase receptor MerTK is involved in several pathophysiological events among which the negative regulation of inflammation and the activation of phagocytosis are the major ones [23,24,26,36,37]. The efficient clearance of apoptotic cells is essential to maintain tissue homeostasis. In particular, this clearance mechanism could help to reduce the development of lesions following persistent injury/inflammation [38]. Therefore, we legitimately hypothesized possible roles of MerTK in regulating some renal I/R-related events and undertook the present study to ascertain such a hypothesis. RCS rats in which a natural mutation of the MerTK receptor gene leads to an inactive truncated MerTK protein are well characterized for a defect of phagocytosis leading to retinal degeneration [32,34,35]. The use of RCS rats as an experimental model for studying the impact of MerTK in renal I/R has not been reported as yet.

In the present report, using for the first time the RCS rat experimental model for studying renal I/R, we investigated major aspects of renal I/R by monitoring changes in plasma- and/or renal tissue- levels of several markers of kidney function and damage such as creatinine, urea, LDH, ASAT, NGAL, and KIM-1. We also quantified the levels of key players of the inflammatory process such MCP-1 and TNF-α, and we examined the recruitment within the renal tissue of CD68-positive inflammatory cells as well as changes in the tissue level of markers of NETosis such as H3a and MPO. Since changes in MPs levels may reflect both the extent of cell damage (production of MPs) or the activation of repair mechanisms (phagocytosis of produced MPs), in parallel complementary experiments, we also investigated possible changes in the plasma levels of total-, platelet-, endothelial- or leucocyte-derived MPs. Furthermore, we set up in vitro phagocytosis assays and monitored the ability of blood-isolated monocytes from WT or RCS rats to phagocytize renal cell apoptotic bodies.

Creatinine and urea levels increased 24 h post-reperfusion in rats but dropped to near baseline on the third day post-reperfusion, suggesting that the renal I/R sequence caused impaired renal function within 1 day followed by a recovery within three days. LDH and ASAT plasma levels followed a similar pattern to that of creatinine and urea. LDH is released from dead cells, and ASAT is an enzyme of the renal tubular epithelial cells, which is released after cellular damage [39]. Therefore, the LDH and ASAT plasma level pattern of the I/R groups suggest a substantial increase in cell damage and necrosis one day post-reperfusion and cell recovery three days post-reperfusion. It should be noted here that an increase in the LDH and ASAT plasma level was also observed in the SHAM groups, but this increase was much lower than that of the I/R groups, thereby confirming that surgery by itself without clamping causes some tissue attrition and cellular damage. NGAL levels increased on day 1 post-reperfusion, and this increase was sustained three days post-perfusion. NGAL is considered a marker of cell stress produced by live damaged tubular epithelial cells and by activated neutrophils but not by necrotic cells [39]. Compared to other markers such LDH or ASAT, data relating to NGAL levels provide additional information, suggesting that following the renal I/R sequence, cellular stress was sustained three days post-reperfusion. KIM-1 blood levels increased in the I/R subgroups one day, and to a greater extent, three days post-perfusion. KIM-1 is expressed by epithelial cells of proximal tubules where it may act as a putative receptor for PS that may contribute to the phagocytic activity of epithelial cells. Oxidative stress activates some metalloproteinases that in turn cleave KIM-1, releasing its ectodomain that can then be detected in the plasma [40]. Therefore, the sustained increase of the KIM-1 plasma level we observed in the I/R subgroup may be part of the repair process of renal lesions.

In addition to biomarker assays, histological analyses revealed diffuse proximal tubule lesions that affected up to 50% of the kidney tissue. Proximal tubules appeared to be the most affected regions, most likely owing to their limited ability to undergo anaerobic metabolism following oxygen depletion [41]. Histological analyses are in agreement with biomarker assays as the renal I/R sequence may lead to increased cell death and accumulation of apoptotic bodies that together with a defect in phagocytosis activity may obstruct the lumen of the tubules and consequently impair renal function (Creatinine and Urea). Finally, as depicted in Figure 3 and Figure 4, the absence of a functional MerTK receptor did not affect the trend but changed the extent of the I/R-induced changes in the plasma levels of some markers such as creatinine and LDH and in renal histological damage.

We next investigated possible changes in the levels of key players of the inflammatory process during the I/R sequence and how these levels may be influenced by MerTK. One important consequence of I/R is monocyte/macrophage infiltration into the injured renal tissue. This infiltration of these phagocytic cells is induced by chemotactic factors produced by injured cells, a major key one is MCP-1, which is a potent chemoattractant protein for monocyte recruitment [42]. The I/R sequence led to a decrease in the expression level of MCP-1 and TNF-α (supplementary data online, Supplementary Figure S2) in renal tissues. However, MCP-1 plasma levels substantially increased one day, and to a greater extent, three days post-I/R, suggesting that renal cells stored and then released MCP-1 after injury. MCP-1 acts by attracting leukocytes to the site of injury and by amplifying the pro-inflammatory loop, in particular via the stimulation of TNF-α production [43,44]. The TNF-α plasma levels could not be quantified as they were below our detection threshold (picograms); supplementary data online, Supplementary Figure S2. Previous studies showed that in rats, TNF-α kidney levels increased during the first 2 h and then decreased at 4 h post-reperfusion, whereas its serum levels peaked 1 h post-reperfusion but were almost nil after that [45].

Since I/R increased the plasma levels of the chemoattractant MCP1, we evaluated the macrophage/neutrophil CD68-positive recruitment into the renal tissue. Our study also demonstrates both the recruitment within the renal tissue of CD68-positive macrophages and neutrophils and the presence of significant renal histological lesions 1 or 3 days post-reperfusion. Our observations are in agreement with previous reports [46,47] and may reflect the requirement within the injured tissue for phagocyte recruitment to clear-up apoptotic bodies and cell debris [38]. Furthermore, our study revealed that the I/R sequence triggered an increase in H3a and MPO levels, suggesting the presence within the renal tissue of activated neutrophils and NETosis [48].

In a second part of our work, we quantified total MPs as well as platelet- (PMPs), endothelial- (EMPs) and leucocyte- (LMPs) derived microparticles in the plasma. We did not observe consistent changes in the level of any of the types of MPs between the various groups and sup-groups studied while previous studies in other experimental models reported significant increases in several MP types following I/R [19,49,50]. Such an apparent discrepancy may be accounted for by differences between some parameters used in the present study as compared to previous ones. These parameters include the animal models used, the duration of the Ischemic phase, the duration post-reperfusion after which blood samples were collected, as well as the cellular origin of the MPs studied.

Finally, we completed our study by several in vitro phagocytosis assays with an aim to evaluate the ability of blood-isolated monocytes (phagocytes) derived from WT or RCS rats to phagocytose NRK-52 cell apoptotic bodies (substrates). For these in vitro phagocytosis assays, we chose to derive apoptotic bodies from the rat kidney epithelial cell line NRK-52 to consider both species and tissue specificity. We chose to expose NRK-52 cells to H_2_O_2_ to derive apoptotic bodies to mimic oxidative stress that may occur during the renal I/R sequence. To set-up this assay, we first conducted preliminary kinetic experiments that enabled us to choose 9-h exposure of isolated monocytes to apoptotic NRK-52 cell bodies as the best duration for a compromise between maximal phagocytosis activity and maximal monocyte survival. In terms of phagocytosis activity, our in vitro phagocytosis assay did not show significant differences between monocytes isolated from WT or RCS rats. It should be noted here that since we aimed at being the closest possible to in vivo conditions, unlike other studies [24], we conducted this phagocytosis assay in the absence of any exogenous addition of MerTK ligand protein S. Nevertheless, exogenous addition of MerTK ligand protein S may be necessary in vitro phagocytosis assay for revealing the extent of MerTK implication in the phagocytosis process. Such an approach may be used in future studies but is beyond the scoop of the present report.

In our RCS rat experiment model and under our experimental settings, the absence of a functional MerTK receptor did not affect the trend but impacted the extent to which the levels of some markers of renal injury, function, and inflammation changed following the I/R sequence. Several hypotheses may explain why the lack of a functional MerTK receptor did not lead to drastic differences between WT and RCS rats following I/R sequences.

First, the absence of functional MerTK may be compensated by the two other members of the TAM receptor family: Axl and Tyro3. Seitz et al. showed that Axl and Tyro3 are critical for the ingestion of apoptotic bodies by dendritic cells (DCs) or macrophages [51]. *Axl*^−/−^, *Tyro3*^−/−^, and *Axl*^−/−^ *Mer*^−/−^ macrophages had ∼40–50% reduction in their ability to phagocytose apoptotic thymocytes. They suggested that interactions between Tyro3, Axl, and MerTK that may form heterodimers occur in macrophages and may be important for their phagocytosis activity [51]. Cell- and organ-type specificity seems to be delineated for phagocytosis activity. Indeed, Mer^−/−^ DCs cells did not demonstrate a significant reduction in the phagocytosis of apoptotic thymocytes, whereas Axl^−/−^, Tyro3^−/−^, and Mer^−/−^ presented a severe deficit in this process [51]. In vitro experiments demonstrated that *Mer*^−/−^ macrophages had a dramatic deficit in phagocytosis of apoptotic thymocytes, but interestingly no significant difference was observed in term of the binding capacities of apoptotic thymocytes by macrophages from wild-type or *Mer*^−/−^ mice. Moreover, a similar phagocytosis activity towards latex beads or opsonized particles by wild-type or *Mer*^−/−^ macrophages was reported [52].

Second, TAM receptor expression is influenced by macrophage polarization [53]. During monocyte to macrophage differentiation, MerTK expression is upregulated while Tyro3 levels remain unchanged [53]. MerTK expression seems to be higher in macrophages M2-like (anti-inflammatory/immunosuppressive phenotype) than in unstimulated or M1-like macrophages [54,55]. There is also a decrease in the anti-inflammatory IL-10 cytokine level and an increase in inflammatory cytokines IL-12 and IL-6 levels in *Mer*^−/−^ mice, yielding a more M1-like macrophage phenotype [55]. Renal ischemia reperfusion induces iNOS-positive proinflammatory (M1) macrophage recruitment in the first few days after reperfusion, whereas macrophages M2-like recruitment predominates at later reperfusion [56]. In our study, we observed an increase in iNOS, which could be in association with a pro-M1 phenotype and consequently a lower MerTK expression. The use of *Mer*^−/−^ DCs or blocking antibodies against MerTK failed to show inhibition of NF-κB activation, suggesting the existence of cross talk or feedback mechanisms between distinct signaling cascades downstream of MerTK regulating phagocytosis and cytokine production [57,58].

The receptor tyrosine kinase MerTK is a major player in both phagocytosis and the negative regulation of pro-inflammatory signaling pathways [36,37,51,59,60]; its possible roles in renal I/R model have not been investigated. Using RCS rats, we developed a renal I/R model that required determining optimal durations of bilateral kidney ischemia. The durations usually used (45 min, 1 h) for other rat strains such as Wistar and Sprague Dawley [61,62,63] lead to a very high percentage of animal mortality. Hence, we conducted the present study with an ischemia duration of 30 min. The effects of I/R were determined on day 1 and day 3 post-reperfusion. It would be interesting to determine in future studies the level of some markers at earlier reperfusion times. For instance, a hepatic I/R study showed an increase in MP levels at earlier times ranging from 1 h to 4 h post-reperfusion [19].

## 4. Materials and Methods

### 4.1. Animals

We used Royal College of Surgeons rats characterized by inherited retinal degeneration that starts at about three weeks of age as retinal pigment epithelial cells fail to phagocytose shed photoreceptor epithelial cells. A causative gene rdy (retinal dystrophy) was identified as a recessive mutation affecting the MerTK gene that models autosomal recessive disease [31,32]. RCS rats are rdy^−/−^ while wild type (WT) are rdy^+/+^. Dystrophic pigmented RCS is referred to here as RCS, and the age-matched non-dystrophic control is referred to here as WT. RCS and WT rats were graciously donated by Dr. E. Nandrot (Institut de la Vision, 17 rue Moreau 75,012 Paris, France) [31] and were bread within our animal facility.

Male rats (12–14 weeks old) weighing 240 and 300 g were used randomly in all experiments. Animal care and experiments were conducted in accordance with the guidelines of the French Agriculture and Forestry Ministry (decree 87849) and of the European Communities Council Directive (86/609/EEC) and were approved by the local ethics committee (COMETHEA: CE2012-06).

### 4.2. Ischemia-Reperfusion Bilateral Kidney Injury

Two experimental groups were set up: RCS rats (RCS rdy^−/−^ not expressing a functional MerTK receptor) and WT rats (expressing functional MerTK) [31,32]. Each group was composed of three subgroups with a minimum of five rats per subgroup. subgroup 1: Control D0 (healthy animals), subgroup 2: I/R D3: 30-min ischemia (by clamping the renal pedicle) with three days of reperfusion, and subgroup 3: SHAM D3: surgery with an identical protocol to the group 2 but without renal pedicle clamping, with three days of reperfusion.

Rats were anesthetized (4% isofluorane for induction and 2% for maintenance). After flank incision, renal pedicles were clamped using micro serrefines (Fine Science Tools, 18055-03, Heidelberg, Germany) for 30 min and then released. The 30′ ischemia duration was determined from preliminary experiments as the duration sufficient to induce kidney injury with severe injuries but with minimal mortality for RCS rats. Collection of blood in Lithium Heparin Tubes (BD Vacutainer, Pont de Claix, France) was performed from the caudal vein seven days before ischemia and one day after reperfusion for biomarker evaluation or phagocytosis test. Three days post-reperfusion, blood was collected in lithium heparin tubes and citrate tubes and kidneys were removed before euthanasia.

### 4.3. Soluble Biomarker Evaluation

Plasma creatinine, plasma urea, plasma LDH, and plasma ASAT were measured seven days before the surgery, and on days 1 and 3 post-surgery to assess renal function and injuries, using the Cobas C701 automatic analyzer (Roche Diagnostic, Basel, Switzerland). TNF-α (RTA00), MCP-1 (DY3144-05), NGAL (DY3508-15), and KIM-1 (DY3689-15) soluble proteins were quantified in plasma by sandwich ELISA (all from R&D Systems, Minneapolis, MI, USA) according to the manufacturer’s instructions.

### 4.4. Western Blot Analyses

Kidney sample were homogenated and incubated in RIPA Buffer (50 mM Tris HCl pH 7.4, 150 mM NaCl, 1% NP-40, 0.5% sodium deoxycholate, 0.1% SDS, 1 mM EGTA, 5 mM EDTA) (Euromedex, Souffelweyersheim, France) with phosphatase and a protease inhibitor cocktail (Sigma-Aldrich, St. Louis, MO, USA) for 1 h at 4 °C. Cell lysates were centrifuged at 9000× *g* for 15 min at 4 °C, and the supernatants were collected and mixed with loading buffer (Tris HCl 50 mM, SDS 70 mM, Glycerol 20%, Bromophenol Blue 1.5 mM, pH 6.8) and heated to 95 °C for 10 min. The proteins were then loaded in a 4–15% gradient polyacrylamide gel (Bio-Rad, 5678084, Hercules, CA, USA). The proteins were transferred, at 25 V for 7 min, on a PVDF membrane (Bio Rad, Hercules, CA, USA) via a Trans-Blot Turbo Transfer System (Bio-Rad, Hercules, CA, USA). This membrane was then saturated in a solution of TBS (TRIS-buffered saline; 250 mM Tris-base, pH 7.4, 1.37 M sodium chloride, 27 mM KCl)-tween 0.1% with non-fat dry milk or bovine serum albumin (BSA) (Supplemental Table S1) and incubated with primary antibody (Supplemental Table S1) overnight at 4 °C. The membrane was then washed five times in TBS-tween 0.1% for 10 min. The proteins recognized by the secondary antibody (Supplemental Table S1) were revealed by bioluminescence using a Luminata solution (Millipore, WBLUF0100, Burlington, MA, USA) with the Bio Rad ChemiDoc MP Imaging System. The relative protein level was normalized to the GAPDH (Glyceraldehyde-3-phosphate dehydrogenase) protein using the ChemiDoc software Image Lab (Bio-Rad, Hercules, CA, USA).

### 4.5. Identification/Quantification MPs

Platelet-poor plasma (PPP) was obtained from blood by two successive centrifugations at 2500× *g*, 20 °C, for 15 min. At each time, the pellet was removed, and the supernatant was collected and stored at −80 °C. The detection of MPs was performed by flow cytometry using the following commercially available antibodies: anti-Mouse/Rat CD61-PE (BioLegend, 104307, 0.2 mg/mL, San Diego, CA, USA), anti-Rat CD54-PE (BioLegend, 202405, 0.2 mg/mL, San Diego, CA, USA), and anti-Rat CD45-V450 (BD Biosciences, 561587, 0.2 mg/mL, Pont de Claix, France), which were used as respective specific anti platelet-derived MPs (PMPs), anti-endothelial-derived MPs (EMPs), and anti-leukocyte-derived MPs (LMPs). For each sample, anti-Mouse/Rat CD61-PE, anti-Rat CD54-PE, and anti-Rat CD45-V450 were used separately in distinct tubes. Their control isotype antibodies (BioLegend, 400907; REF isotype control CD54, San Diego, CA, USA; BD Biosciences, 561504, Pont de Claix, France) were used in parallel experiments to estimate non-specific signals; 30 µL of PPP was incubated with 10 µL Annexin-V-FITC (BD Biosciences, 556419, Pont de Claix, France) and 10 µL of antibodies prediluted (anti-Mouse/Rat CD61-PE: 1/20; anti-Rat CD54-PE: 1/10; anti-Rat CD45-V450: 1/20) for 30 min in the dark at room temperature. After incubation, 500 µL of Annexin-V buffer (BD Bioscience, 556454) with 2 ATU/mL Hirudin (Sigma-Aldrich, H0393-100UN, St. Louis, MO, USA) and 30 µL MP Count Beads (Stago, 01169, Asnières-sur-Seine, France) were added. The microparticles were detected using a BD FACS Verse flow cytometer (BD Biosciences, Pont de Claix, France) equipped with three lasers at 405, 488, and 633 nm (configuration 4-2-2).

Prior to PPP acquisition, the cytometer was calibrated using a mix of fluorescent beads of different diameters, selected to cover a major part of the theoretical MP size range (0.1 to 1 µm), using SSC as a size-related parameter (Megamix Plus-SSC, Biocytex, Marseille, France) (Figure 6b). Bead acquisition according to the manufacturer’s instructions allows setting the cytometer to study MP within a constant size region. This size MP gate (Figure 6a) was applied during the analysis of cell-specific MPs (Figure 6c,e,g,i). Each PPP was acquired in the presence of MP Count Beads to determine the concentration of MPs. An unlabeled sample was acquired to detect the sample auto-fluorescence and set the negative population. The use of multiple fluorochromes in one acquisition may cause their waveforms to overlap of their emission waves. Fluorescent cross-talk was controlled by compensation adjustment. Compensation settings were established by acquiring single-color stained tubes. The use of the isotype control allows us to detect unspecific stained samples. Figure 6c,e,g,i show an example of the analysis of cell-specific MPs. The number of double-positive MPs was determined to deduce the concentration of this population in the PPP. The analyses were performed using FlowJo v10 software.

### 4.6. Histopathology Analyses

Kidneys were collected and immediately fixed in 4% formol, embedded in paraffin, and sectioned at 3 µm thickness. Histological Periodic-acid-Schiff (PAS) coloration was performed to evaluate tubular injury [64]. Several criteria were assessed: tubular dilatation, tubular necrosis, interstitial inflammation, cell detachment, loss of brush border, intracellular oedema, and exposed basal membrane. All criteria followed an injury scale of 1 to 5: 1 (no damage), 2 (damage affecting less than 10% of the whole kidney section), 3 (damage affecting 25% of the whole kidney section), 4 (damage affecting 50% of the whole kidney section), and 5 (damage affecting 75% or more of the whole kidney section). For immunohistochemistry, the sections were heated for 30 min at 100 °C in Tris-EDTA buffer (Tris 1.21 g/L; EDTA 0.37 g/L, tween 0.3%, pH 9) for antigen retrieval. Then the immunohistological staining was performed using the monoclonal mouse anti-CD68 antibody (Bio-Rad; MCA341GA, Hercules, CA, USA). Examination and quantification were performed using Leica DM4000 B LED with a “Leica DFC 295” camera and the associated “Leica Application Suite” software for imaging in a blinded fashion.

### 4.7. Phagocytosis Test In Vitro

Phagocytosis of CMF-DA-labeled apoptotic NRK-52 cells by blood monocytes from RCS and WT rats was assessed by flow cytometry and a confocal microscope. CMF-DA (5-chloromethylfluorescein diacetate) (Abcam; ab145459, Cambridge, United Kingdom) is a dye that freely crosses cell membranes and is then processed and retained within the cell. Briefly, CMF-DA-labeled apoptotic NRK-52 cells were obtained by incubating NRK-52 with DMEM containing 5 µM CMF-DA for 30 min at 37 °C. Then, cells were incubated with DMEM containing Fetal Bovine Serum (FBS) 2% containing sodium bicarbonate 1% + H_2_O_2_ 100 µM to induce apoptosis induced by oxidative stress. Blood monocytes were isolated from WT and RCS rat blood. Red blood cells were lysed with lysis buffer (NH4Cl0.15 M; KHCO3 10 mM; Na2EDTA; 0.1 mM; pH 7.2–7.4). After centrifugation at 400× *g* for 5 min, the blood leukocyte pellet was seeded in a 1% gelatin flask and incubated overnight at 37 °C and 5% CO_2_. The next day, the flask supernatant (containing non-adherent cells) was removed and the flask was washed several times with 1X Phosphate Buffer Saline (PBS) to remove the remaining red blood cells and lymphocytes, leaving only adherent cells (monocytes) on the flask. For phagocytosis assay, CMF-DA-labeled apoptotic NRK-52 cells were exposed to blood monocytes (collected from healthy animal WT and RCS): target ratio 1:5 with RPMI FBS 10% for 9 h at 37 °C. At the end of the phagocytosis assay, monocytes were collected and labeled with a phycoerythrin anti-CD45 (BioLegend; 202207, San Diego, CA, USA) and incubated in 2 mg/mL trypan blue (Invitrogen) to quench the fluorescence of surface-bound non-engulfed fluorescent material. Results were examined using an FACS BD Acuri C6 (BD Biosciences, Pont de Claix, France) and analyzed using FlowJo Software. The percentage of monocytes that had CMF-DA corresponded to cells that had ingested labeled NRK-52 apoptotic fragments. We used compensation and FMO controls for the flow experiment as described in Supplementary Figure S3. For confocal microscopy, the phagocytosis assay was performed on a slice. Monocytes were labeled with a phycoerythrin anti-CD45 and examined using an Olympus FV1000 confocal microscope. As a positive control, 2 µm fluorescent beads were used.

### 4.8. Statistical Analysis

The data were presented as the mean ± SEM using R-studio software. Statistical analyses, involving more than two groups, were performed using the Kruskal–Wallis test followed by Dunn’s test to obtain a comparison between each group. For statistical analysis between two groups, the Mann–Whitney test was used. Significant differences were *p* > 0.05: ns (not significant); *p* < 0.05: *; *p* < 0.01: **; *p* < 0.005: ***.

## 5. Conclusions

In conclusion, the present report describes the development of a renal I/R model for the RCS rat strain with the monitoring of a wide range of renal function, renal tissue, and plasma markers that paves the way for further investigation of MerTK- dependent events in renal tissue injury and repair mechanisms.

## Data Availability

The data presented in this study are available on request from the corresponding author.

## Supplementary Materials

The following are available online at https://www.mdpi.com/article/10.3390/ijms222212103/s1.

## Abbreviations

ADAM-17	ADAM metallopeptidase domain 17
ASAT	Aspartate aminotransferase
BSA	Bovine serum albumin
DAMPs	Damage-associated molecular patterns
DCs	Dendritic cells
EMPs	Endothelial-derived microparticles
FACS	Fluorescence-activated single cell sorting
H3A	Histone 3A
IL	Interleukin
iNOS	inducible nitric oxide synthase
I/R	Ischemia-reperfusion
KIM-1	Kidney injury molecule -1
LDH	Lactate dehydrogenase
LMPs	Leukocyte-derived microparticles
MCP-1	Monocyte chemoattractant protein-1
MPs	Microparticles
MPO	Myeloperoxidase
NET	Neutrophil extracellular traps
NGAL	Neutrophil gelatinase-associated lipocalin
NO	Nitric oxide
PAS	Periodic-acid-Schiff
PMPs	Platelet-derived microparticles
PPP	Platelet-poor plasma
PS	Phosphatidylserine
ROS	Reactive oxygen species
RCS	Royal College of Surgeons
TAM	Tyro3, Axl and MerTK family
TLR	Toll-like receptors
TNFα	Tumor necrosis factor-α

## References

[1] Chiang-Ting C., Tzu-Ching C., Ching-Yi T., Song-Kuen S., Ming-Kuen L. (2005). Adenovirus-Mediated Bcl-2 Gene Transfer Inhibits Renal Ischemia/Reperfusion Induced Tubular Oxidative Stress and Apoptosis. Am. J. Transplant..

[2] Eltzschig H.K., Eckle T. (2011). Ischemia and Reperfusion—From Mechanism to Translation. Nat. Med..

[3] Favreau F., Giraud S., Bon D., Chatauret N., Thuillier R., Hauet T. (2013). Ischemia reperfusion control: The key of kidney graft outcome. Med. Sci..

[4] Pefanis A., Ierino F.L., Murphy J.M., Cowan P.J. (2019). Regulated Necrosis in Kidney Ischemia-Reperfusion Injury. Kidney Int..

[5] Wiegele G., Brandis M., Zimmerhackl L.B. (1998). Apoptosis and Necrosis during Ischaemia in Renal Tubular Cells (LLC-PK1 and MDCK). Nephrol. Dial. Transplant..

[6] Zindel J., Kubes P. (2020). DAMPs, PAMPs, and LAMPs in Immunity and Sterile Inflammation. Annu. Rev. Pathol..

[7] Munkonda M.N., Akbari S., Landry C., Sun S., Xiao F., Turner M., Holterman C.E., Nasrallah R., Hébert R.L., Kennedy C.R.J. (2018). Podocyte-Derived Microparticles Promote Proximal Tubule Fibrotic Signaling via P38 MAPK and CD36. J. Extracell. Vesicles.

[8] Brinkmann V., Zychlinsky A. (2012). Neutrophil Extracellular Traps: Is Immunity the Second Function of Chromatin?. J. Cell Biol..

[9] Liu J., Dong Z. (2018). Neutrophil Extracellular Traps in Ischemic AKI: New Way to Kill. Kidney Int..

[10] Nakazawa D., Kumar S.V., Marschner J., Desai J., Holderied A., Rath L., Kraft F., Lei Y., Fukasawa Y., Moeckel G.W. (2017). Histones and Neutrophil Extracellular Traps Enhance Tubular Necrosis and Remote Organ Injury in Ischemic AKI. J. Am. Soc. Nephrol..

[11] Pacher P., Beckman J.S., Liaudet L. (2007). Nitric Oxide and Peroxynitrite in Health and Disease. Physiol. Rev..

[12] Huen S.C., Cantley L.G. (2015). Macrophage-Mediated Injury and Repair after Ischemic Kidney Injury. Pediatric Nephrol..

[13] Huen S.C., Cantley L.G. (2017). Macrophages in Renal Injury and Repair. Annu. Rev. Physiol..

[14] Škrajnar Š., Lasnik M.A., Zavec A.B. (2009). A Flow Cytometric Method for Determination of the Blood Neutrophil Fraction in Rats. J. Am. Assoc. Lab. Anim. Sci..

[15] Buzas E.I., György B., Nagy G., Falus A., Gay S. (2014). Emerging Role of Extracellular Vesicles in Inflammatory Diseases. Nat. Rev. Rheumatol..

[16] Mobarrez F., Svenungsson E., Pisetsky D.S. (2018). Microparticles as Autoantigens in Systemic Lupus Erythematosus. Eur. J. Clin. Investig..

[17] Vallier L., Cointe S., Lacroix R., Bonifay A., Judicone C., Dignat-George F., Kwaan H.C. (2017). Microparticles and Fibrinolysis. Semin. Thromb. Hemost..

[18] Mohning M.P., Thomas S.M., Barthel L., Mould K.J., McCubbrey A.L., Frasch S.C., Bratton D., Henson P.M., Janssen W.J. (2017). Phagocytosis of Microparticles by Alveolar Macrophages during Acute Lung Injury Requires MerTK. Am. J. Physiol. Lung Cell Mol. Physiol..

[19] Freeman C.M., Quillin R.C., Wilson G.C., Nojima H., Johnson B.L., Sutton J.M., Schuster R.M., Blanchard J., Edwards M.J., Caldwell C.C. (2014). Characterization of Microparticles after Hepatic Ischemia-Reperfusion Injury. PLoS ONE.

[20] Shao W.-H., Zhen Y., Rosenbaum J., Eisenberg R.A., McGaha T.L., Birkenbach M., Cohen P.L. (2010). A Protective Role of Mer Receptor Tyrosine Kinase in Nephrotoxic Serum-Induced Nephritis. Clin. Immunol..

[21] Fraineau S., Monvoisin A., Clarhaut J., Talbot J., Simonneau C., Kanthou C., Kanse S.M., Philippe M., Benzakour O. (2012). The Vitamin K-Dependent Anticoagulant Factor, Protein S, Inhibits Multiple VEGF-A-Induced Angiogenesis Events in a Mer- and SHP2-Dependent Manner. Blood.

[22] Tondo G., Perani D., Comi C. (2019). TAM Receptor Pathways at the Crossroads of Neuroinflammation and Neurodegeneration. Dis. Markers.

[23] Ginisty A., Gély-Pernot A., Abaamrane L., Morel F., Arnault P., Coronas V., Benzakour O. (2015). Evidence for a Subventricular Zone Neural Stem Cell Phagocytic Activity Stimulated by the Vitamin K-Dependent Factor Protein S: Phagocytic Activity of Neural Stem Cells. Stem Cells.

[24] Barth N.D., Marwick J.A., Heeb M.J., Gale A.J., Rossi A.G., Dransfield I. (2018). Augmentation of Human Monocyte Responses to Lipopolysaccharide by the Protein S and Mer/Tyro3 Receptor Tyrosine Kinase Axis. J. Immunol..

[25] Burger D., Schock S.C., Thompson C.S., Montezano A.C., Hakim A.M., Touyz R.M. (2013). Microparticles: Biomarkers and Beyond. Clin. Sci..

[26] Cohen P.L., Shao W.-H. Gas6/TAM Receptors in Systemic Lupus Erythematosus. https://www.hindawi.com/journals/dm/2019/7838195/.

[27] Cohen P.L., Caricchio R., Abraham V., Camenisch T.D., Jennette J.C., Roubey R.A.S., Earp H.S., Matsushima G., Reap E.A. (2002). Delayed Apoptotic Cell Clearance and Lupus-like Autoimmunity in Mice Lacking the c-Mer Membrane Tyrosine Kinase. J. Exp. Med..

[28] Lu Q., Gore M., Zhang Q., Camenisch T., Boast S., Casagranda F., Lai C., Skinner M.K., Klein R., Matsushima G.K. (1999). Tyro-3 Family Receptors Are Essential Regulators of Mammalian Spermatogenesis. Nature.

[29] Duncan J.L., LaVail M.M., Yasumura D., Matthes M.T., Yang H., Trautmann N., Chappelow A.V., Feng W., Earp H.S., Matsushima G.K. (2003). An RCS-like Retinal Dystrophy Phenotype in Mer Knockout Mice. Investig. Opthalmol. Vis. Sci..

[30] Camenisch T.D., Koller B.H., Earp H.S., Matsushima G.K. (1999). A Novel Receptor Tyrosine Kinase, Mer, Inhibits TNF-Alpha Production and Lipopolysaccharide-Induced Endotoxic Shock. J. Immunol..

[31] Nandrot E.F., Dufour E.M. (2010). Mertk in Daily Retinal Phagocytosis: A History in the Making. Adv. Exp. Med. Biol..

[32] D’Cruz P.M., Yasumura D., Weir J., Matthes M.T., Abderrahim H., LaVail M.M., Vollrath D. (2000). Mutation of the Receptor Tyrosine Kinase Gene Mertk in the Retinal Dystrophic RCS Rat. Hum. Mol. Genet..

[33] LaVail M.M., Battelle B.A. (1975). Influence of Eye Pigmentation and Light Deprivation on Inherited Retinal Dystrophy in the Rat. Exp. Eye Res..

[34] Nandrot E., Dufour E.M., Provost A.C., Péquignot M.O., Bonnel S., Gogat K., Marchant D., Rouillac C., de Condé B.S., Bihoreau M.T. (2000). Homozygous Deletion in the Coding Sequence of the C-Mer Gene in RCS Rats Unravels General Mechanisms of Physiological Cell Adhesion and Apoptosis. Neurobiol. Dis..

[35] Parinot C., Nandrot E.F. (2016). A Comprehensive Review of Mutations in the MERTK Proto-Oncogene. Adv. Exp. Med. Biol..

[36] Dransfield I., Zagórska A., Lew E.D., Michail K., Lemke G. (2015). Mer Receptor Tyrosine Kinase Mediates Both Tethering and Phagocytosis of Apoptotic Cells. Cell Death Dis..

[37] Zhen Y., Finkelman F.D., Shao W.-H. (2018). Mechanism of Mer Receptor Tyrosine Kinase Inhibition of Glomerular Endothelial Cell Inflammation. J. Leukoc. Biol..

[38] Arandjelovic S., Ravichandran K.S. (2015). Phagocytosis of Apoptotic Cells in Homeostasis. Nat. Immunol..

[39] Zager R.A., Johnson A.C.M., Becker K. (2013). Renal Cortical Lactate Dehydrogenase: A Useful, Accurate, Quantitative Marker of In Vivo Tubular Injury and Acute Renal Failure. PLoS ONE.

[40] Han W.K., Bailly V., Abichandani R., Thadhani R., Bonventre J.V. (2002). Kidney Injury Molecule-1 (KIM-1): A Novel Biomarker for Human Renal Proximal Tubule Injury. Kidney Int..

[41] Sheridan A.M., Bonventre J.V. (2000). Cell Biology and Molecular Mechanisms of Injury in Ischemic Acute Renal Failure. Curr. Opin. Nephrol. Hypertens..

[42] Sung F.L., Zhu T.Y., Au-Yeung K.K.W., Siow Y.L., O K. (2002). Enhanced MCP-1 Expression during Ischemia/Reperfusion Injury Is Mediated by Oxidative Stress and NF-KappaB. Kidney Int..

[43] Akcay A., Nguyen Q., Edelstein C.L. (2009). Mediators of Inflammation in Acute Kidney Injury. Mediat. Inflamm..

[44] Lawrence T., Natoli G. (2011). Transcriptional Regulation of Macrophage Polarization: Enabling Diversity with Identity. Nat. Rev. Immunol..

[45] Meldrum K.K., Meldrum D.R., Meng X., Ao L., Harken A.H. (2002). TNF-Alpha-Dependent Bilateral Renal Injury Is Induced by Unilateral Renal Ischemia-Reperfusion. Am. J. Physiol. Heart Circ. Physiol..

[46] Chen X., Wang C.-C., Song S.-M., Wei S.-Y., Li J.-S., Zhao S.-L., Li B. (2015). The Administration of Erythropoietin Attenuates Kidney Injury Induced by Ischemia/Reperfusion with Increased Activation of Wnt/β-Catenin Signaling. J. Formos. Med. Assoc..

[47] Li L., Okusa M.D. (2010). Macrophages, Dendritic Cells, and Kidney Ischemia-Reperfusion Injury. Semin. Nephrol..

[48] Yu Y., Kwon K., Tsitrin T., Bekele S., Sikorski P., Nelson K.E., Pieper R. (2017). Characterization of Early-Phase Neutrophil Extracellular Traps in Urinary Tract Infections. PLoS Pathog..

[49] Faure V., Dou L., Sabatier F., Cerini C., Sampol J., Berland Y., Brunet P., Dignat-George F. (2006). Elevation of Circulating Endothelial Microparticles in Patients with Chronic Renal Failure. J. Thromb. Haemost..

[50] Teoh N.C., Ajamieh H., Wong H.J., Croft K., Mori T., Allison A.C., Farrell G.C. (2014). Microparticles Mediate Hepatic Ischemia-Reperfusion Injury and Are the Targets of Diannexin (ASP8597). PLoS ONE.

[51] Seitz H.M., Camenisch T.D., Lemke G., Earp H.S., Matsushima G.K. (2007). Macrophages and Dendritic Cells Use Different Axl/Mertk/Tyro3 Receptors in Clearance of Apoptotic Cells. J. Immunol..

[52] Scott R.S., McMahon E.J., Pop S.M., Reap E.A., Caricchio R., Cohen P.L., Earp H.S., Matsushima G.K. (2001). Phagocytosis and Clearance of Apoptotic Cells Is Mediated by MER. Nature.

[53] Myers K.V., Amend S.R., Pienta K.J. (2019). Targeting Tyro3, Axl and MerTK (TAM Receptors): Implications for Macrophages in the Tumor Microenvironment. Mol. Cancer.

[54] Spanuth E., Breyer J. (1988). Experiences with a Statistical Quality Control System (QCS) for Coagulation Diagnostics Parameters. Folia Haematol..

[55] Grabiec A.M., Goenka A., Fife M.E., Fujimori T., Hussell T. (2018). Axl and MerTK Receptor Tyrosine Kinases Maintain Human Macrophage Efferocytic Capacity in the Presence of Viral Triggers. Eur. J. Immunol..

[56] Lee S., Huen S., Nishio H., Nishio S., Lee H.K., Choi B.-S., Ruhrberg C., Cantley L.G. (2011). Distinct Macrophage Phenotypes Contribute to Kidney Injury and Repair. J. Am. Soc. Nephrol..

[57] Rothlin C.V., Ghosh S., Zuniga E.I., Oldstone M.B.A., Lemke G. (2007). TAM Receptors Are Pleiotropic Inhibitors of the Innate Immune Response. Cell.

[58] Sen P., Wallet M.A., Yi Z., Huang Y., Henderson M., Mathews C.E., Earp H.S., Matsushima G., Baldwin A.S., Tisch R.M. (2007). Apoptotic Cells Induce Mer Tyrosine Kinase-Dependent Blockade of NF-KappaB Activation in Dendritic Cells. Blood.

[59] Lee Y.-J., Han J.-Y., Byun J., Park H.-J., Park E.-M., Chong Y.H., Cho M.-S., Kang J.L. (2012). Inhibiting Mer Receptor Tyrosine Kinase Suppresses STAT1, SOCS1/3, and NF-ΚB Activation and Enhances Inflammatory Responses in Lipopolysaccharide-Induced Acute Lung Injury. J. Leuko. Biol..

[60] Zhen Y., Priest S.O., Shao W.-H. (2016). Opposing Roles of Tyrosine Kinase Receptors Mer and Axl Determine Clinical Outcomes in Experimental Immune-Mediated Nephritis. J. Immunol..

[61] Fan L., He L., Cao Z., Xiang B., Liu L. (2012). Effect of Ischemia Preconditioning on Renal Ischemia/Reperfusion Injury in Rats. Int. Braz. J. Urol..

[62] Ko S.-F., Yip H.-K., Zhen Y.-Y., Lee C.-C., Lee C.-C., Huang S.-J., Huang C.-C., Ng S.-H., Lin J.-W. (2017). Severe Bilateral Ischemic-Reperfusion Renal Injury: Hyperacute and Acute Changes in Apparent Diffusion Coefficient, T1, and T2 Mapping with Immunohistochemical Correlations. Sci. Rep..

[63] Kocoglu H., Ozturk H., Ozturk H., Yilmaz F., Gulcu N. (2009). Effect of Dexmedetomidine on Ischemia-Reperfusion Injury in Rat Kidney: A Histopathologic Study. Ren. Fail..

[64] Goujon J.M., Hauet T., Menet E., Levillain P., Babin P., Carretier M. (1999). Histological Evaluation of Proximal Tubule Cell Injury in Isolated Perfused Pig Kidneys Exposed to Cold Ischemia. J. Surg. Res..

