# Supplementary material for: Study of the Role of the Tyrosine Kinase Receptor MerTK in the Development of Kidney Ischemia-Reperfusion Injury in RCS Rats
Source: Int J Mol Sci. 2021 Nov 9;22(22):12103. doi: 10.3390/ijms222212103 (PMC8618874; doi:10.3390/ijms222212103)
Supplement: Supplementary file 1 [file ijms-22-12103-s001.zip › ijms-1227215-supplementary.pdf]

Supplementary Materials

Supplemental Methods:

Rat Genotyping: Biopsies were performed on RCS rat tails. DNA extraction was performed with the REDExtract-N-Amp Tissue PCR Kit (Sigma; XNAT-100RXN) according to the manufacturer's instructions. Polymerase Chain Reaction (PCR) was performed on the Rotor-Gene Q Lightcycler (Qiagen) using the 2X PCR Mix from the REDExtract-N-Amp Tissue PCR Kit and 500 nM of the specific primer for rat MerTK, forward: TGCTGAAAAGGAGGAAGAAATCAAG, and reverse: AATGATGTGCCCCAAGCAGT. Samples were loaded in Agarose gel 2%—SyBR Safe 1X (Invitrogen; S33102). DNA PCR products were visualized using the ChemiDoc MP imaging system (Bio-Rad).

Supplemental Figures and Table:

Table S1. Antibodies used for Western Blotting experiments.

Target protein	Manufacturer	References	Host	Dilution	Blocking Buffer	Size (kDa)
MerTK	FabGennix	MKT-101AP	Rabbit	1/1000^{e}	BSA	150
MCP-1	Biovision	5225-100	Rabbit	1/1000^{e}	Milk	20
iNOS	BD Biosciences	610431	Mouse	1/1000^{e}	BSA	130
TNF-α	Abcam	Ab6671	Rabbit	1/1000^{e}	Milk	25
H3a	Abcam	Ab5103	Rabbit	1/2000^{e}	BSA	17
CD68	Bio-Rad antibodies.com	MCA341GA	Mouse	1/1000^{e}	Milk	25
MPO	Abcam	Ab208670	Rabbit	1/1000^{e}	Milk	59
Anti-rabbit-HRP	Abcam	Ab6721	Goat	1/1000^{e}	Milk	
Anti-mouse-HRP	Abcam	Ab6789	Goat	1/1000^{e}	Milk	

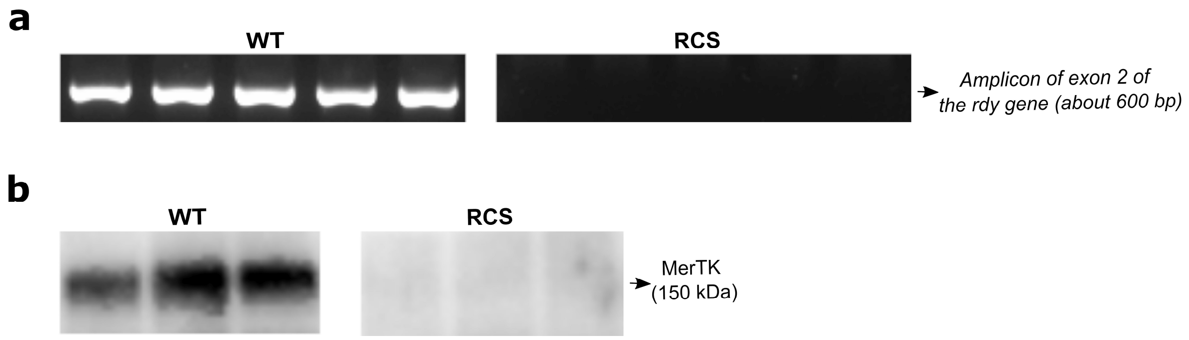

Figure S1. MerTK is not expressed in RCS rats. In panel a, DNA extracted from both WT or RCS rats were analysed by PCR and electrophoresis gel for the presence of mutation on RCS rats gene *rdy (MER)*. In panel b, kidney tissue lysates from both WT or RCS rats were analysed by western blotting for the presence of MerTK protein (150 kDa).

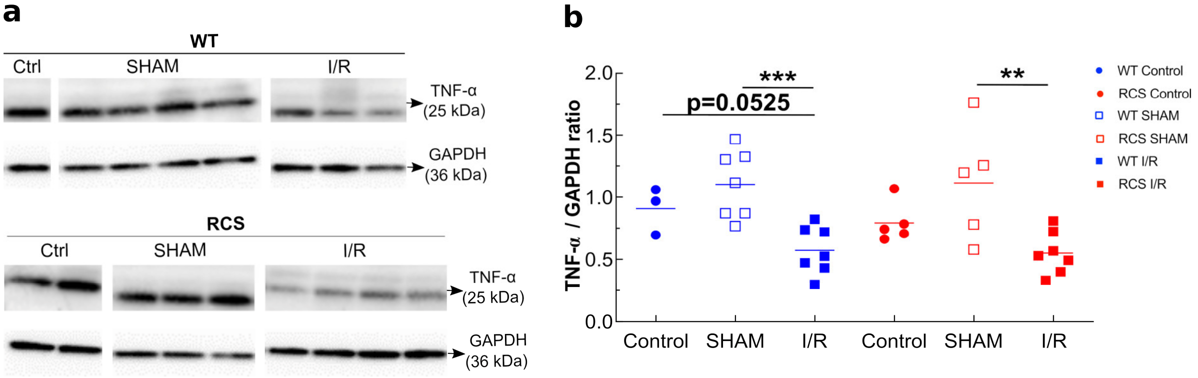

Figure S2. Kidney levels of TNF-α in both WT[+] and RCS rats in control, SHAM and I/R conditions at day 1 or day 3 post-reperfusion. In panel **a**, kidney tissue lysates from both WT or RCS rats that were either not submitted to surgery (Control), submitted to surgery without renal pedicle clamping (SHAM) or with a 30 min renal pedicle clamping followed by 3 days reperfusion (I/R), were analyzed by western blotting for the presence of TNF-α protein (25 kDa). Total protein loaded are shown in the bottom of each western blot. In panel **b**, the intensity of TNF-α bands were normalized to total protein load and are represented, 3 to 8 rats were used for each condition. The Mann-Whitney test was used for determining the p values. *: $p<0.05$; **: $p<0.01$; ***: $p<0.005$.

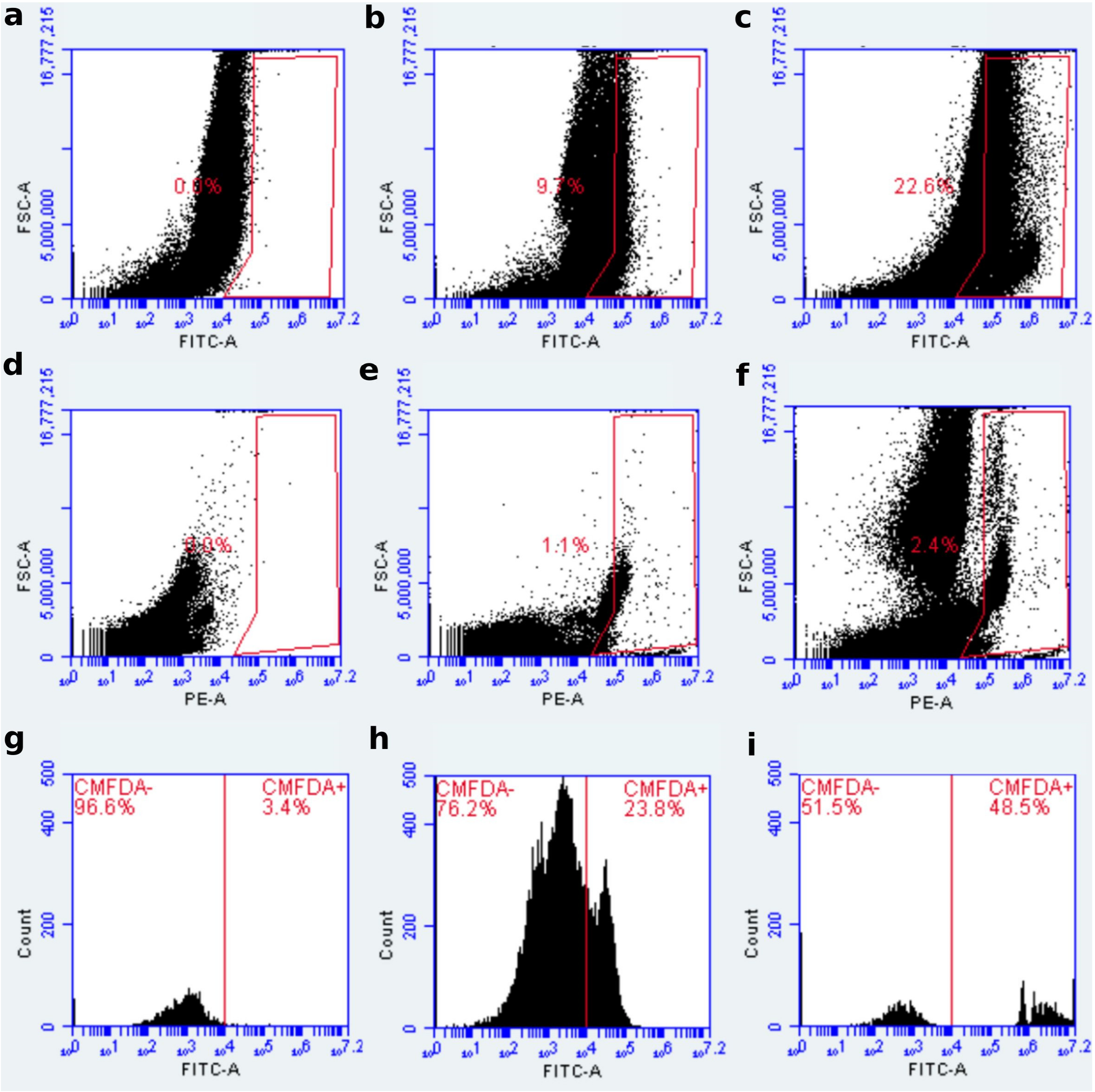

Figure S3. Gating strategy of In vitro phagocytosis activity by flow cytometry. Apoptotic bodies were obtained from cultured rat kidney cells (NRK-52 cells) that were exposed to 100 M H_2O_2 for 3 hours. NRK-52 cell apoptotic bodies were labelled with CMF-DA green tracker and used in the phagocytosis assay. Monocytes isolated from either RCS or WT rat blood samples were exposed to either NRK-52 cell apoptotic bodies or to fluorescent latex beads (positive control) for 9 h. Monocytes were then labelled with fluorescent (red) anti-CD45 antibody and their ability to bind or to internalize either NRK-52 cell apoptotic bodies or fluorescent latex beads was analyzed by flow cytometry using a FACS BD Acuri C6. The % of phagocytosis is determined by the following gating strategy : a Unlabeled NRK-52 cells; b Annexin V-FITC-labelled NRK-52; c CMF-DA-labelled NRK-52; d Unlabeled monocytes; e CD45-PE-labelled monocytes; f CD45-PE-labelled monocytes + CMF-DA-labelled NRK-52; g CMF-DA negative population defined from CD45 positive population; h Example of representation showing CD45 positive and CMF-DA positive cells; i Positive control (fluorescent beads. (n=6).
